# Coaching the coaches: exploring the effectiveness of the ‘Move Well Be strong’ youth injury prevention programme for grassroot coaches and PE teachers

**DOI:** 10.1080/07853890.2024.2408456

**Published:** 2024-09-27

**Authors:** J. D. Hughes, F. Ayala, W. M. Roberts, K. Wing, M. B. A. De Ste Croix

**Affiliations:** a Youth Physical Development Center, Cardiff School of Sport and Health Sciences, Cardiff Metropolitan University, Cardiff, UK; b School of Natural, Social and Sport Sciences, University of Gloucestershire, Gloucester, England, UK; c Department of Sport Sciences, University of Murcia, Murcia, Spain; d Te Huataki Waiora - School of Health, University of Waikato, Hamilton, New Zealand

**Keywords:** Youth, injury prevention, coach education, RE-AIM framework

## Abstract

**Introduction:**

Coaches play a major role in developing movement in their performers, especially at grassroots levels. However, there are significant knowledge gaps amongst grassroots coaches and physical education (PE) teachers regarding movement competency and injury prevention programs. This study aimed to explore the effectiveness of knowledge gain, adoption and implementation following a youth injury prevention workshop for grassroots coaches and PE teachers.

**Methods:**

56 grassroots coaches and PE teachers completed a validated questionnaire exploring use, knowledge, attitude towards and confidence to deliver youth movement competency training before and after an online workshop. Bayesian Wilcoxon signed-rank tests were used to assess the knowledge, attitude, and confidence to deliver an injury prevention programme following the workshop. For all the Bayesian inference tests run, the Bayesian factor (BF_10_) was interpreted using the evidence categories ranging from extreme evidence (BF_10_ > 100) to anecdotical evidence (BF_10_ < 1).

**Results:**

Post-workshop there was a 34% increase in respondents indicating that they had greater knowledge of injury prevention issues (55% pre-workshop vs 89% post-workshop) with statistically positive and moderate effects (BF_10_ > 100 [extreme evidence]). There was also a 25% increase in respondents indicating that they had a more sympathetic attitude towards injury prevention (67% sympathetic pre-workshop vs 93% sympathetic post-workshop) with statistically moderate effects (BF_10_ = 87.4 [very strong evidence]). A 19% increase in attendees’ confidence to deliver an injury prevention programme was observed (69% high pre-workshop vs. 89% high post-workshop) with statistically moderate effects (BF_10_ = 85.9 [very strong evidence]). 100% of participants indicated an intent to adopt the injury prevention programme.

**Conclusions:**

An online workshop increased knowledge and confidence in grassroots coaches and PE teachers to deliver a youth injury prevention programme. Knowledge gained from training and upskilling created a positive attitude and confidence to deliver movement competency into coaching. Appropriate resources need to be developed and delivered in an accessible way to grassroots coaches and PE teachers *via* workshops and should be included in governing body coaching awards or as continuing professional development for youth coaches and PE teachers.

## Introduction

Children who have previously been sedentary and then started physical activity (PA) and sport are at a greater risk of sustaining an injury than those who have participated in sport from a young age [1]. However, irrespective of the sport that children are participating in, it is well recognized that children aged between 12–18yr are at the greatest risk of sustaining a serious non-contact injury that has both short and long-term health consequences [2]. Evidence highlights that there is high risk of paediatric sport injury which contributes significantly to public health expenses [3,4]. A concern regarding long-term consequences of youth sports injury is the risk of developing osteoarthritis (OA) at a young age. Based on the available evidence, a link between youth sports injuries, particularly acute injury of the knee and ankle, and OA, is likely [5].

Sport Coaches (SC) and Physical Education (PE) teachers are key to encouraging and ensuring that children in PE classes and sport settings adopt appropriate safe practices [6]. However, the extent to which SC and PE teachers undertake this role is influenced by their knowledge, beliefs, and attitude towards injury prevention programmes [7]. Injury prevention programmes need to be age, sex, and maturation specific with clear progressions as a child grows and matures. There is currently a need to develop such materials that are suitable for grassroots coaches and PE teachers, founded on principles of fun and high levels of engagement in these settings. It is well recognized that coaches who uptake and adhere to such prevention programmes can reduce injury incidence in their youth athletes by up to 80% [8] and one randomized control trial saw an 89% reduction in injury rates during just one season of the adoption of an injury prevention programme [9]. Further, evidence establishes children who demonstrate good levels of motor skill competency in sport and PE generally have more fun, greater self-esteem and stay engaged in physical activity, leading to a greater overall health status of the nation [10]. Coupled to growing evidence for the efficacy of injury prevention programmes, evidence of significant challenges to implementing these programmes has emerged [11]. So, despite the well-recognized benefits of adopting an injury prevention programme, uptake, adherence, and compliance are often poor [11]. This is concerning as high compliance has been associated with greater injury reductions. SC have been identified as important adoption targets for injury prevention programmes in amateur soccer, but recent studies have identified low levels of amateur coaches using such programmes. Linked to these data are significant knowledge gaps amongst community level coaches regarding injury prevention programmes [12,13]. Currently there is very little data focusing on PE teachers, which is surprising given the daily teaching of children in a sport and physical activity context.

Given children are at high-risk of serious non-contact sporting injuries, with both short and long term associated health issues, they therefore are an important target group for injury prevention programmes. While elite young athletes have access to sports science and medical support through their clubs, it is often the responsibility of grassroots coaches and PE teachers to care for and ensure the well-being of the children they instruct and coach, since many young people who participate in sports do not do so at an elite level [14]. The effectiveness of the implementation of injury prevention programmes are often assessed *via* the RE-AIM framework (Reach, Efficacy, Adoption, Implementation and Maintenance) [11]. A recent systematic review conducted on the RE-AIM framework for injury prevention programs, as acknowledged by O’Brien and Finch[11], revealed significant gaps in terms of the adoption and maintenance of these programs. A study by Steffen et al. [15] investigated differences in the delivery of knowledge to coaches and explored subsequent adherence to an injury prevention programme. The study introduced coaches to the FIFA 11+ either *via* an unsupervised website, a coach focused workshop with (“Comprehensive”) and without (“Regular”) additional physiotherapy support. Adherence to the programme was significantly greater where a workshop was delivered and was equally successful with or without additional support, as opposed to just a web-based programme. These data reinforce the need for coach education programmes focusing on injury prevention for youth to be delivered *via* a workshop method but with subsequent access to online resources. In one of the few studies on such programmes in school setting [16] noted that key barriers to successful implementation were the complexity of the programme (with the need to limit the number of components and equipment required), and lack of readiness for implementation. Key facilitators included adaptability of the school but most importantly a positive implementation climate and culture. Data from Barden et al. [17,18], also in school settings, illustrates the importance of adapting programs to meet the needs of PE teachers based on their setting and context. The ability to adapt materials and new knowledge appears to be important in the success of implementing the programme. Combined, these data support the key aspect of organizational buy-in as essential for successful implementation in both PE and sport settings.

The management and delivery of coach education and continuing professional development (CPD) for registered coaches are typically handled by the National associations/federations. National strategies are created to establish general awards and CPD for coaches, which are then typically implemented by regional associations. Teacher education is commonly provided by universities, with a strong emphasis on understanding and safeguarding the needs of the child. Despite this focus, there often lacks a provision of CPD workshops or materials on youth injury prevention programs that are integral to player well-being. Given the crucial role of grassroots coaches and PE teachers in promoting athlete well-being—a significant part of their responsibilities—there is a clear need, as Bennett et al., [19] suggest, for the development of a dedicated workshop. Such a workshop, along with associated materials, would equip grassroots youth sports coaches and PE teachers with the necessary skills and knowledge to confidently deliver movement competency programmes. The main aim of this project is to upskill grassroots SC and PE teachers to deliver effective movement competency programmes, to enhance the well-being of youths involved in sport and physical education.

## Methods

### Participants

Ninety-five participants (28 females and 67 males; mean age 37.5 ± 11.2 y) who were grassroots SC and/or PE teachers from all regions in Saudi Arabia attended an online workshop, (Move Well, Be Strong). In total 82% of participants were either qualified PE teachers and/or had coaching qualifications. Some grassroots SC and PE teachers worked with multiple age groups with the total numbers (n) working with specific age groups presented as: U7–9 = 22, U10–12 = 27, U13–16 = 28, 17 y+ = 33. All participants (*n* = 95) completed a pre-workshop questionnaire, with *n* = 56 (60%) completing the post workshop questionnaire and *n* = 14 (25% of post participants) completing the follow-up questionnaire. There were certain inclusion and exclusion criteria that needed to be met by the participants. To be included, the participant had to be willing and able to give informed consent for participation, be either male or female aged 18 years or above and have participated in the Move Well Be Strong online workshop delivered through Microsoft Teams and remained in attendance for the duration of the workshop. Written informed consent was provided by completing an online form with a digital signature. On the other hand, participants were excluded from the project if they did not coach or teach youth players. The study was performed in accordance with the Declaration of Helsinki and received approval from the University of Gloucestershire Research Ethics Committee (DESTECROIX21-22(2)).

### Questionnaires

SCs’ and PE teachers’ knowledge of, attitude towards, and confidence to deliver youth injury prevention as part of their coaching and PE lessons were explored at the start and end of the workshop *via* a validated online questionnaire (using SurveyHero) (see additional material for questionnaires). An online questionnaire was sent out to all those who completed the post-workshop questionnaire 2-4 months’ post completion of the workshop to explore the adoption, implementation, and maintenance of the programme. The questionnaires were compiled following the Reach, Effectiveness, Adoption, Implementation and Maintenance (RE-AIM) framework [20] after a review of the wider coach education literature, and in collaboration with a local advisor in Saudi Arabia to ensure that any country-specific issues were addressed. The framework has been mostly applied as an evaluation tool but has broader applications as a planning tool and as a method to review intervention studies [21]. The scales, items and concepts deployed were derived and adapted, in part, from the survey employed by De Ste Croix et al. and O’Brien and Finch [7,11] exploring the perceptions of the deliverers of injury prevention training in grassroots coaches. Following pilot testing, the final set of questions were developed and agreed upon through consultation between the authors, and an external panel of experts. The first part of each questionnaire elicited demographic and background information from participants including the level of coaching qualification, sex, age group coached/taught, number of years coaching/teaching. The second part of the questionnaire assessed 10 questions related to knowledge of injury prevention programmes, attitude towards injury prevention and confidence to deliver injury prevention. Perceived barriers and facilitators towards delivering such training were also explored. These were assessed both in terms of the relative level of importance attributed to each item (rated on a 5–point scale ranging from Strongly Agree (5) to Strongly Disagree (1). The questionnaires were administered in both English and Arabic.

A number of measures were put into place to try and maximize the response rates to the surveys including: (a) offering the questionnaires in both English and Arabic (b) time given to complete questionnaire 1 and 2 during the online session; (c) direct emailing of the web link to Questionnaire 1 prior to the online workshop for completion; (d) following up with numerous reminders to non-respondents; (e) providing a strong rationale for the project and describing the value we place in participants’ responses; (f) ensuring the surveys were of high quality and easy to read, and (g) ensuring that the surveys were as simple as possible to complete without missing important data needed to address the project objectives (h) receiving and providing to participants a letter of support from the Saudi Ministry for Sport.

### Workshop

The workshop delivered online, consisted of a theory session (35 min) that included information on injury incidence and risk factors in youth sport, the influence of growth and maturation of injury risk, and programmes for injury prevention including their effectiveness. This was followed by a practical session (60 min) delivering the **Move Well, Be Strong** project movement competency components. The practical session was created and delivered by experienced practitioners who had extensive experience in coaching and working with children. The programme was centred on the Athletic Motor Skills Competencies (AMSC) [22]. The central tenet of the programme was to allow the SC and PE teachers to visualize the exercise, practice the exercises themselves then be given no more than three external cueing examples for them to focus on when delivering the programme [23]. Animations and descriptions of all the movements, and a digital manual, were available to participants on a dedicated website post-workshop (www.movewellbestrong.com). The workshop was delivered online ‘live’ using the Mevo Start 3 camera system (Mevo Inc. 19 Morris Avenue, Brooklyn, NY) which allowed multiple angles to be shown during the practical session.

### Statistical analysis

Descriptive data are presented as percentage values (%) and exclude incomplete answers to questions. Where a five-point Likert scale was used responses 4 and 5 (agree/strongly agree) were used to show agreement to that question. Statistical analyses were performed using JASP (Amsterdam, Netherland) software version 0.10. The potential effects elicited by the workshop on SC and PE teachers’ knowledge, attitude, and confidence to deliver an injury prevention programme were assessed using separate Bayesian Wilcoxon signed-rank tests.

For all the Bayesian inference tests run, the Bayesian factor (BF_10_) was interpreted using the evidence categories previously suggested [24] < 1/100 = extreme evidence for H_0_; from 1/100 to <1/30 = very strong evidence for H_0_; from 1/30 to <1/10 = strong evidence for H_0_; from 1/10 to <1/3 = moderate evidence for H_0_; from 1/3 to <1 anecdotical evidence for H_0_; from 1 to 3 = anecdotical evidence for H_1_; from >3 to 10 = moderate evidence for H_1_; from >10 to 30 = strong evidence for H_1_; from >30 to 100 = very strong evidence for H_1_; >100 extreme evidence for H_1_.

The median and the 95% central credible interval (CI) of the posterior distribution of the standardized effect size (δ) (i.e. the population version of Cohen’s d) were also calculated for each of the paired comparisons carried out. Magnitudes of the posterior distribution of the standardized effect size were classified as: trivial (<0.2), small (0.2–0.6), moderate (0.6–1.2), large (1.2–2.0) and very large (2.0–4.0) [25].

Separate Bayesian binomial tests were conducted to assess whether the category proportion of the dichotomous variables obtained after the workshop were equal (null hypothesis [H_0_]) to the test value (presumed population value = 0.5). Furthermore, separate Bayesian multinomial tests were also run to analyze whether the category proportions in polytomous variables were uniformly distributed (null hypothesis [H_0_]).

## Results

Table 1 displays participants’ knowledge, attitude, and confidence before and after the injury prevention workshop.

**Table 1.** Participants’ knowledge, attitude, and confidence before and after the injury prevention workshop.

Ordinal labels	Before workshop		After workshop	
	n	%	N	%
Knowledge of injury prevention issues				
1. Very poor	3	5.4	0	0
2. Poor	2	3.6	0	0
3. Not good / not poor	20	35.7	6	10.7
4. Good	13	23.2	12	21.4
5. Very good	18	32.1	38	67.9
Attitude toward injury prevention				
1. Indifferent	0	0	0	0
2. A bit Indifferent	2	3.6	0	0
3. Not sympathetic / not indifferent	16	28.6	4	7.1
4. A bit sympathetic	11	19.6	10	17.9
5. Sympathetic	27	48.2	42	75.0
Confidence to deliver an injury prevention programme				
1. Very low	0	0	0	0
2. Low	6	10.7	0	0
3. Not high / not low	11	19.6	6	10.7
4. High	13	23.2	12	21.4
5. Very high	26	46.4	38	67.9

**Figure 1.**
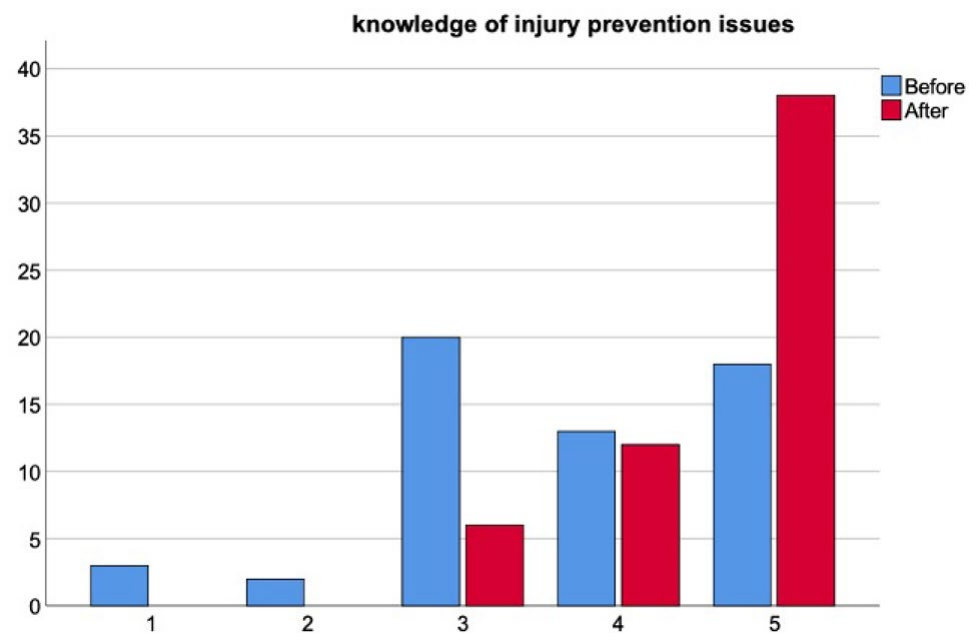
Injury prevention knowledge at the start and end of the workshop.

**Figure 2.**
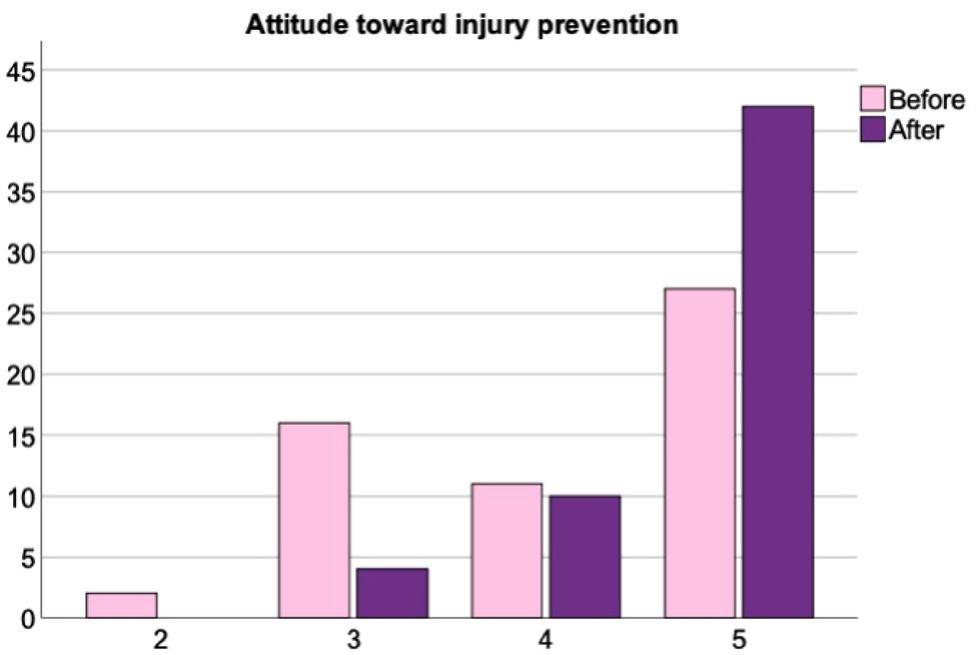
Attitude towards youth injury prevention training at the start and end of the workshop.

**Figure 3.**
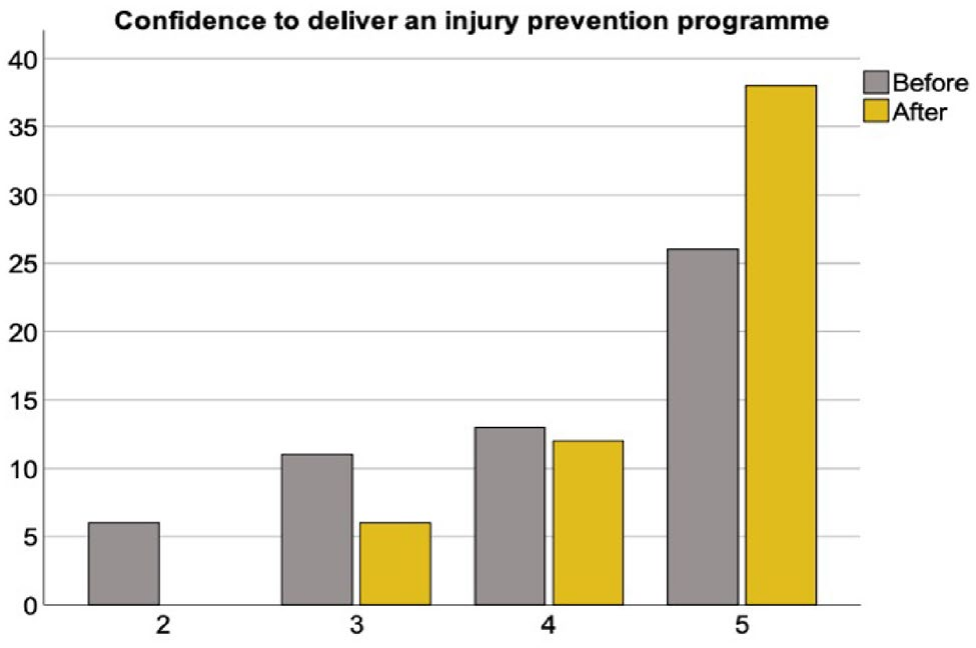
Confidence to deliver injury prevention training at the start and end of the workshop.

The workshop elicited positive and moderate effects on participants knowledge of (BF_10_ > 100 [extreme evidence in favor of H_1_], *W* = 28, Rhat = 1.01 [convergence], δ = 0.79 [95% IC = from 0.48 to 1.12]) and attitude toward (BF_10_ = 87.4 [very strong evidence in favor of H_1_], *W* = 14, Rhat = 1 [convergence], δ = 0.59 [95% IC = from 0.29 to 0.91]) injury prevention (figures 1 and 2). Furthermore, participants’ confidence to deliver an injury prevention programme was also significantly increased (BF_10_ = 85.9 [very strong evidence in favor of H_1_], *W* = 6.5, Rhat = 1 [convergence], δ = 0.58 [95% IC = from 0.27 to 0.91]) (Figure 3).

### Knowledge of youth injury prevention

Prior to the workshop some participants knowledge regarding injury prevention was very good/good (55%). Post workshop 89% of coaches rated their knowledge as very good/good, a 34% increase. These data can be seen in Figure 1 below.

### Attitude towards youth injury prevention

Prior to the workshop, nearly a third of coaches’ and PE teachers’ attitudes (32%) towards injury prevention training with their youth players was either poor or indifferent. From the start to the end of the workshop coaches and PE teachers with a very good and good attitude towards injury prevention training increased from 68% to 93% (a 25% increase). These data can be seen in Figure 2 below.

### Confidence to deliver youth injury prevention training

31% of coaches and PE teachers did not feel confident to deliver injury prevention training to youth players as part of their everyday coaching or teaching prior to the workshop. However, by the end of the workshop 89% of coaches and teachers felt confident (high and very high confidence) to integrate injury prevention training into their coaching and teaching. These data can be seen in Figure 3 below.

### Intention to adopt, implement and maintain

Nearly all participants (96%) felt that the workshop provided them with the knowledge to deliver youth injury prevention training and 90% of coaches felt satisfied with the workshop. 100% of coaches indicated that they would use the material and knowledge from the workshop and associated websites to deliver injury prevention to their youth athletes. The importance of coaches and physical education teachers receiving training to help them build confidence in delivering movement competency training was acknowledged by 98% of the participants. Bayesian analysis indicated extreme evidence for accepting the alternative hypothesis for all questions. These data can be seen in Table 2 below:

**Table 2.** Participants’ willingness to adopt and maintain the material following the injury prevention workshop.

Question	*n*	%	Bayesian factor (BF_10_)
Do you think you will use injury prevention information in your sessions?*			
Yes	56	100	
No	0	0	
How satisfied are you with the workshop? / My satisfaction with the workshop is^Τ^			
1.	0	0	>100 (extreme evidence for H_1_)
2.	0	0	
3.	6	10.7	
4.	8	14.3	
5.	42	75	
To what extent do you think you will be able to adapt the materials / information to suit my needs^Τ^			
1.	0	0	>100 (extreme evidence for H_1_)
2.	0	0	
3.	5	8.9	
4.	12	21.4	
5.	39	69.6	
To what extent do you think you will use the materials / information in all of my sessions^Τ^			
1.	0	0	>100 (extreme evidence for H_1_)
2.	0	0	
3.	5	8.9	
4.	12	21.4	
5.	39	69.6	
The materials / information will be useful in the long term^Τ^			
1.	0	0	>100 (extreme evidence for H_1_)
2.	0	0	
3.	6	10.7	
4.	5	8.9	
5.	45	80.4	
Do you think there is need for training to help people feel more confident about delivering injury prevention? ^Τ^:			
Yes	55	98.2	>100 (extreme evidence for H_1_)
No	0	0	
Do not know	1	1.8	
Overall do you think the workshop provided you with the knowledge to use injury prevention?*:			
Yes	54	96.4	>100 (extreme evidence for H_1_)
No	2	3.6	

H_1_: alternative hypothesis; H_0_: null hypothesis; *: statistical inference obtained from a Bayesian binomial test; ^Τ^: statistical inference obtained from a Bayesian multinomial test.

### Adoption, implementation, and adherence to injury prevention training

**Figure 4.**
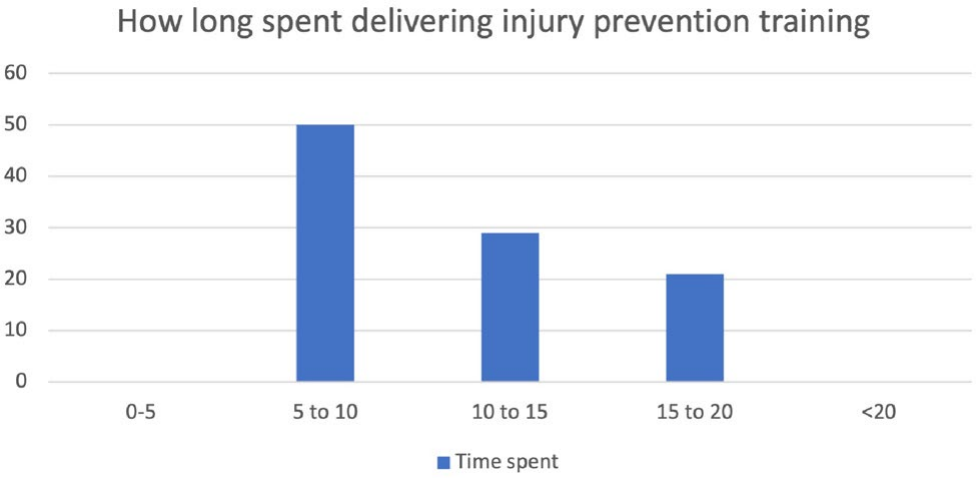
Time spent delivering injury prevention training at training sessions.

All coaches and PE teachers who were followed up 3–4 months after the workshop had adopted the programme (100%) and all were still using the programme as a measure of maintenance. In terms of implementation, most coaches were using their new knowledge at every coaching session or PE lesson (79%). The most common amount of time dedicated to the movement competency exercise was 5-10mins per session (50%) with only 21% of participants using exercises for 15-20min. The time spent delivering injury prevention training can be seen in Figure 4 below. 93% of participants had used or were continuing to use the materials on the website, including animations and the digital manual. Most coaches and PE teachers had shared their new knowledge with others (86%).

## Discussion

Our data suggests that a bespoke and well-designed youth injury prevention workshop can significantly increase knowledge, attitude, and confidence of grassroot coaches and PE teachers to deliver such programmes. Given the data showing that low numbers of coaches are either aware of injury prevention programmes, ranging from 22–27% [26,27], or using these programmes (15–23%) [7,26], workshops to develop knowledge and confidence to deliver such programmes seem essential. However, evaluating their effectiveness is key to determining the impact on knowledge gain, confidence, and intent to adopt. The effectiveness of the current workshop is evident in the significant large effects on knowledge gain, which resulted in a 34% increase (from 55% to 89%). This is very similar to the 33% increase in knowledge gain observed in the study of Russomano et al. [28] which was also delivered 100% online [29] have reported significantly greater increases in knowledge gain, and this might have been attributed to the face-to-face nature of the delivery, and the lower level of knowledge pre workshop. The current workshop was specifically designed for grassroot 2020 coaches and teachers to make sure that the language used, and the level of technical demonstration was suitable for those coaches/teachers. This knowledge gain translated into a statistically significant improvement in attitude towards injury prevention training (25% increase) and importantly confidence to deliver such training (20% increase). These data are similar to the findings of O’Conner and Lacey [26] and De Ste Croix et al. [29] who reported a significant improvement in attitude towards injury prevention training following a face-to-face workshop. Likewise, the 89% of coaches and teachers indicating they were more confident to deliver injury prevention training in the present study is similar to the 89% found by O’Conner and Lacey [26] and 85% of De Ste Croix et al. [29]. Further, the inclusion of practical sections in both the current workshop and those of O’Conner and Lacey [26] and De Ste Croix et al. [29] may have contributed to the increased confidence in coaches to deliver the programme to their populations. Although knowledge gain, confidence, and intent to implement are important characteristics in the development of coaches they do not show that behaviour change has taken place. The study of Frank et al. [30] indicated that high levels of coach intent following an injury prevention workshop did not translate into effective implementation. Conflicting data are available and a recent study in 41 school rugby coaches showed that delivering a workshop increased adoption rates [17]. The development of the Move Well, Be Strong workshop and associated materials provided grassroot youth sport coaches and PE teachers with the skills and knowledge to be able to confidently deliver an injury prevention programme. This knowledge gain is important in terms of adoption, implementation and maintenance and translated into a 100% intent to adopt the programme.

### Adoption

The adoption rate in the current study (100%) is excellent and might be attributed to the time taken by the research team to develop, adapt, and pilot the materials with grassroot coaches and coach educators. This adoption rate is higher than that reported by De Ste Croix et al. [29] (86%), O’Conner and Lacey [26] (73%) and Frank et al. [30] (53%). In contrast to Frank et al. [30] paper there was a good transference from intention to use to adoption in the current study. It appears that the knowledge gain, positive attitude, and confidence to deliver such training that was accrued by coaches and teachers *via* the online workshop translated into a change in behaviour. This supports recent work by Barden et al. [18], where they evidenced that face-to-face workshops increased adoption rates. Our findings must be viewed with a degree of caution as there might be some selection bias where only coaches and teachers who had adopted the programme completed the follow-up questionnaire. This is despite a reasonably good follow-up sample size of 25% which is comparable with the 27% reported by De Ste Croix et al. [29]. Barriers and facilitators to adoption, implementation and maintenance should be explore further using qualitative methods, especially in complex social and cultural setting.

One of the unexpected outcomes of the workshop was the development of a Community of Practice (CoP), with 86% of participants sharing their new knowledge with others. Lark [31] explained the critical role knowledge, its retainment and subsequent sharing plays as a recourse for the development of organizations. Both engagement and attitude are important in the development and sharing of knowledge. The frequent mention of interaction with peers as a key source of knowledge development supports the notion that CoP are key for learning and knowledge sharing [32].

### Implementation

The implementation of the programme was highly successful, with 79% of coaches incorporating elements of it either in every session or at least once a week for 5–15 min, which is almost identical to the data found in the study of De Ste Croix et al. [29] on European sports coaches. Implementation in other studies has been more variable and this might be attributed to the very prescriptive nature of the injury prevention programme (IPP). Most IPPs tend to replace the traditional warm-up, however, Move Well, Be Strong was designed specifically not to be a replacement of the warm-up but rather coaches and teachers were encouraged to introduce exercises throughout their sessions within both drills and games (games formed a part of the post workshop digital manual). Further exploration is needed to determine if the level of flexibility that grassroot coaches and teachers appreciated was a contributing factor to the increased implementation rates. Likewise, it is likely that the practical elements of the workshop were favourable for this level of coach/teacher and this hypothesis is supported by the work of Ling et al. [33] who reported greater implementation with a practical session incorporated into the delivery of the workshop. Our data would seem to support the view that designing the right ‘toolkit’ for the level of coach and teacher is important for successful implementation of the programme. Frank et al. [30] reported lower implementation levels when it came to correcting poor movement compared with completing the whole programme delivered and would suggest that there may be a disparity in the way coaches approach correcting poor movement versus completing a prescribed programme. Specifically, coaches may be more likely to follow a set of instructions rather than focus on the nuances of movement, which could explain the lower implementation rates reported in the study. To expand on this idea, it is important to consider the role of coaching in promoting optimal movement patterns. While completing a prescribed programme is an important part of coaching, it is equally important to pay close attention to the details of movement and adjust as necessary to help athletes achieve optimal performance. However, coaches may be more likely to follow a prescribed set of instructions rather than deviate from it to address specific issues with an athlete’s movement. This tendency to follow a prescribed set of instructions may be driven by several factors, including a desire to stay within the confines of a specific programme or to avoid making mistakes when correcting an athlete’s movement. Additionally, coaches may feel more comfortable focusing on the broader aspects of a programme rather than getting bogged down in the details of individual movements. Taken together, these factors may help explain why coaches may be more likely to complete a prescribed programme rather than correct poor movement, which in turn could account for the lower implementation rates reported in the study by Frank e al. [30]. Ultimately, it is important for coaches to strike a balance between following a programme and paying attention to the finer details of movement to ensure that athletes can achieve their full potential.

Given that both coaching knowledge and coaching age are lower in grassroots coaches it is likely they feel more confident in delivering material rather than correcting poor movement (‘complete’ vs ‘correct’). This reinforces conclusions made by Arundale et al. [34] who stated that in most sports, the coach is key for implementation and compliance, especially amongst nonelite and youth athletes. Thus, a lack of coaching awareness emphasizes the importance of improving the knowledge translation from national sport federations to local sports clubs, using appropriate materials and language. It may be that perceived barriers to implementation might be buy-in from organizations as well as parents, especially in hierarchical cultural environments. It should be noted that the current study is part of a series of studies where we have explored the barriers and facilitators to AIM using qualitative methods to explore individuals’ experiences.

### Maintenance

It is likely that repetitive warm up type IPPs may become boring over time and thus reduce the maintenance of such programmes. This is evident in the study of Silvers-Granelli et al. [35] who reported that when compliance to an injury prevention programme is high, there is a significant reduction in injury and time loss. O’Conner and Lacey [26] recently reported maintenance rates of 73% but this was only 4 weeks after completion of the workshop. The maintenance rates in the current study, 3 months post the workshop, were excellent with all coaches and teachers still using the programme (100%). One of the key elements of the programme was to reinforce to coaches and teachers the fun element of the movements and the incorporation of these into game play type activities. It is possible that allowing coaches to introduce the movements into fun aspects of the training sessions, rather than as a substitute warm up, helped with the maintenance rates. This would support the data from Shamlaye et al. [36] who noted that maintenance was greater in coaches when they were encouraged to adapt exercises. Importantly the Move Well Be Strong programme is not overly prescribed (e.g. no set number of sets or reps) which allows coaches to implement in the ways that works for their children, and this allows variety which possibly resulted in the good maintenance rates. These data reinforce the need for coach education programmes focusing on injury prevention for youth to be delivered *via* a workshop method but with subsequent access to online resources. Our data support the effectiveness of face-to-face workshops as the adoption, implementation and maintenance rates were high. Our findings suggest that the additional materials available to coaches and PE teachers after the workshop are crucial for good maintenance, but organizations may wish to consider whether the resource investment in terms of time and money to develop such supplementary resources are necessary.

#### Practical application

This programme is effective in developing knowledge and confidence to deliver movement competency training in both PE and coaching settings and should be implemented as part of national governing bodies CPD activities or more effectively embedded into coaching awards. It should also form part of the PE curriculum for trainee teachers in higher education settings. To enhance the reach of the programme a comprehensive/enhanced programme aimed at educating the coach educators to deliver these workshops is needed. Good surveillance data is needed to see if the translation of the programme from the coaches to the children in terms of injury risk reduction is evident. Given the importance of access to additional resources in terms of maintenance of the programme the development of more resources, and providing more examples of how the movements could be embedded within a games-based approach is needed. Currently, there is no such systematic data collection in PE and coach settings in youth sports in Saudi Arabia and this is needed to examine both the extent of the injury problem in Saudi Arabia and to explore the efficacy of the programme.

#### Limitations

Although the response rate to our follow-up questionnaires is in line with most follow-up studies (25%), we appreciate that such data may be susceptible to respondent bias where only those who adopted the programme responded. Future research should attempt to investigate the barriers to adoption in those who did not start using the programme. Although we had differences in the sample size between sexes (due to the smaller population of PE teachers and coaches in Saudi Arabia) the Bayesian approaches we took to data analysis allowed us to account for the unbalanced data sets. However, future studies should continue to examine females who are a hard-to-reach group within the Saudi Arabian context, but there is a growing number of female PE teachers since girls PE was introduced in 2020. We acknowledge that the maintenance phase was relatively short (3 months post workshop) and further work should investigate the efficacy of such programmes in terms of long-term maintenance. Future studies should also explore the fidelity of a movement competency education programme by utilizing qualitative methods to explore both the barriers and facilitators to adoption, implementation, and maintenance.

## Conclusions

In conclusion, the workshop on youth movement competency training for injury prevention was effective in increasing knowledge, changing attitudes, and providing confidence for coaches and PE teachers to deliver the training. Most coaches who attended were satisfied with the workshop and all coaches expressed their intention to adopt the new knowledge into their practice. The follow-up survey revealed that all coaches who responded had indeed adopted the programme and maintained using it for several months after the workshop. Furthermore, the fact that most coaches shared their new knowledge with colleagues demonstrates a community of practice effect. Overall, these findings suggest that the workshop had a positive impact on the coaches’ knowledge, attitudes, and behaviour, and it provides a promising model for promoting injury prevention among young athletes.

## Data Availability

Data related to this manuscript will be provided based on reasonable request to the corresponding author.
